# Supplementary material for: Electroluminescence from multi-particle exciton complexes in transition metal dichalcogenide semiconductors
Source: Nat Commun. 2019 Apr 12;10:1709. doi: 10.1038/s41467-019-09781-y (PMC6461636; doi:10.1038/s41467-019-09781-y)
Supplement: Supplementary file 1 — Supplementary Information [file 41467_2019_9781_MOESM1_ESM.pdf]

Supplementary Information

Electroluminescence from multi-particle exciton complexes in transition metal dichalcogenide semiconductors

Paur et al.

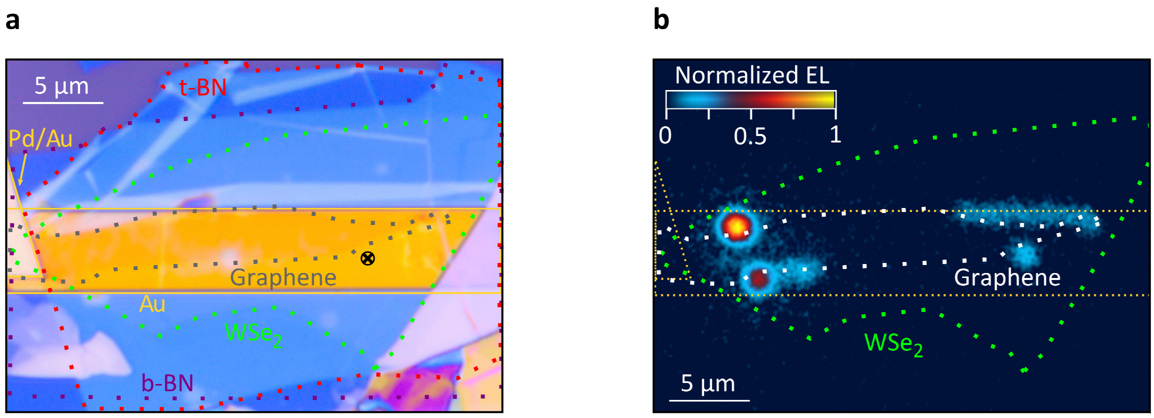

Supplementary Figure 1 | Electroluminescence image of WSe_2 sample at 300 K. a, Optical microscopy image of the sample. **b,** False-color EL image of the sample. The EL emission is generated near the interface between graphene and monolayer WSe_2. However, the light emission is not homogenously generated along this interface, because part of the graphene/WSe_2 junction is not gated and possibly also because of an inhomogeneous contact between graphene and WSe_2. Also note that in this particular device the WSe_2 flake bends over the bottom electrode edge, facilitating the detection of the emission from dark excitons with out-of-plane dipole (see Figs. 2c and 2e in the main text).

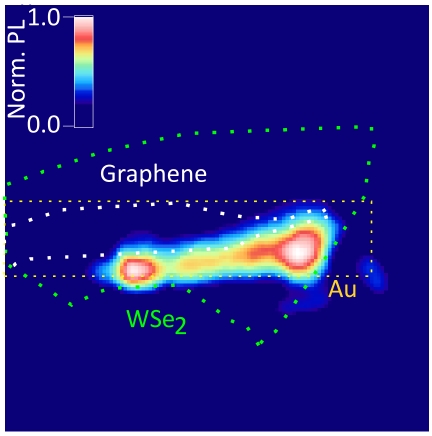

Supplementary Figure 2 | Photoluminescence map at 300 K (WSe_2 sample). Photoluminescence from monolayer WSe_2 at room temperature (excitation $\lambda =$ 532 nm, $P_d =$ 8×10^3 W cm^{-2}). The PL is quenched on graphene.

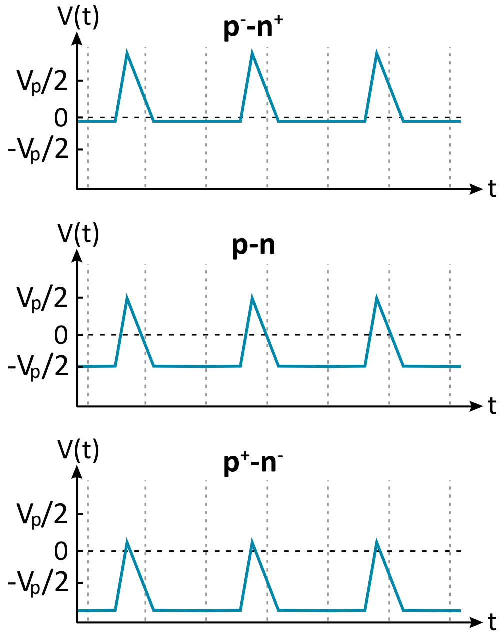

Supplementary Figure 3 | Schematic pulse diagrams. Top: electron-rich (p^-/n^+); Middle: balanced (p/n); Bottom: hole-rich (p^+/n^-) carrier injection.

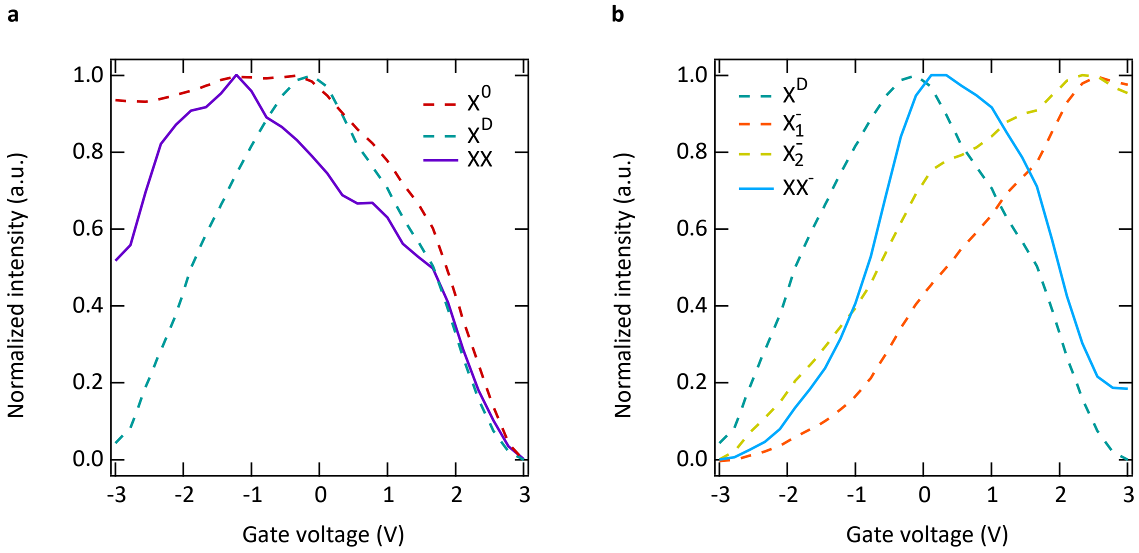

Supplementary Figure 4 | PL emission from exciton complexes as a function of external electric field in WSe_2. Line-cut of the normalized PL intensity of different exciton species. **a,** Biexcitons (purple) **b,** charged biexcitons (blue) as a function of doping level. The behavior of the different peaks with the back voltage is in good agreement with recently reported results[1–4].

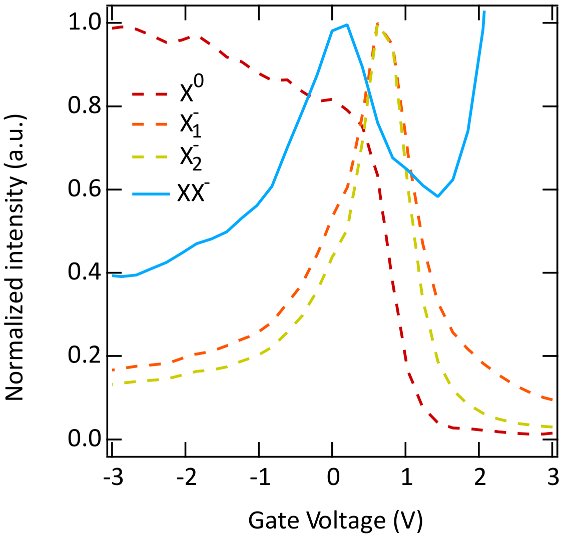

Supplementary Figure 5 | PL emission from exciton complexes as a function of external electric field in WS_2. Line-cut of the normalized PL intensity of different exciton species as a function of doping level. In the highly n-doped regime ($V_G > 2$ V), where X^0 (red), X_1^- (orange), X_2^- (yellow), XX^- (blue) species vanish, the next charging state of the trion may be observed, as in WSe_2[3].

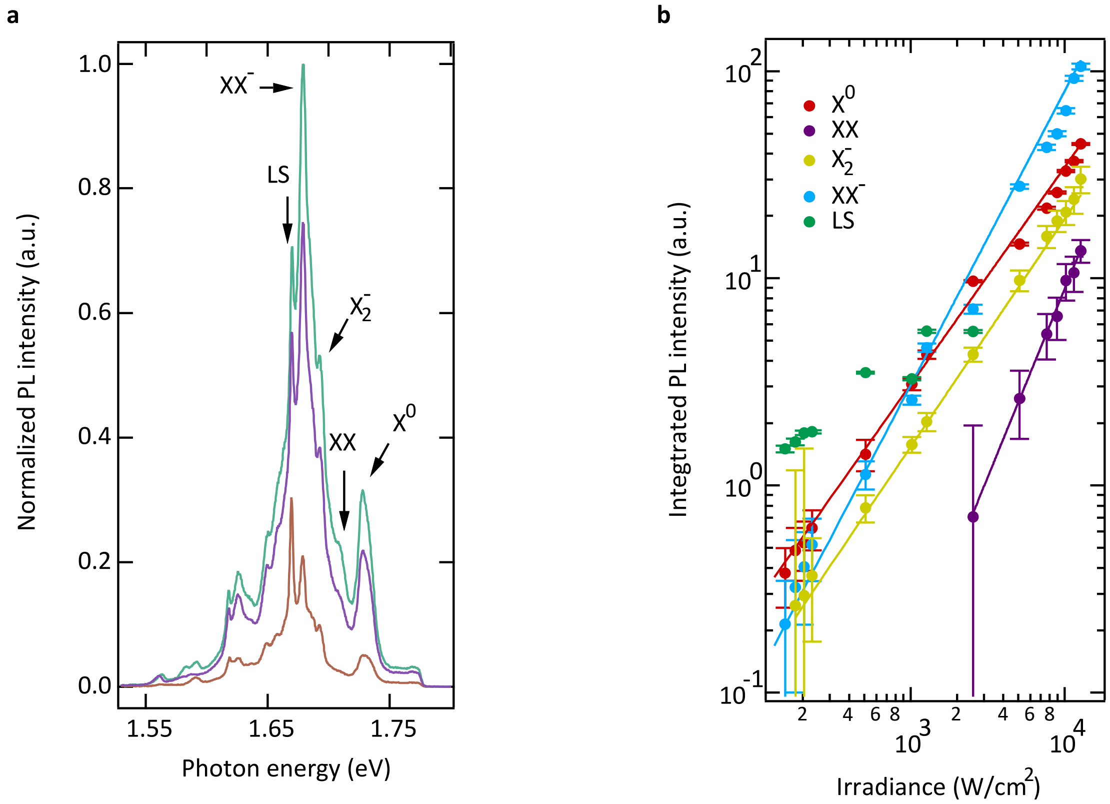

Supplementary Figure 6 | Power-dependence of the PL intensity in WSe_2. a, PL spectra of a WSe_2 monolayer for different excitation intensities (P_d = 2.5x10^3 Wcm^{-2} to 1.2x10^4 Wcm^{-2}). **b,** Logarithmic plot of the integrated PL intensity as a function of the excitation intensity. Circles represent the experimental data, while lines

represent a power law fit, $\propto P^{\alpha}$, with exponents of X^0 (α = 1.01), XX (α = 1.82), X_2^- (α =1.10), XX^- (α = 1.44). The peak LS (α = 0.6), centered at an energy of 1.6696 eV, is most probably due to a localized state, since the dependence on excitation intensity is sublinear[5–10]. The power-dependence measurements are performed without gate voltage (intrinsic regime) at the position marked with a cross in Supplementary Figure 1a.

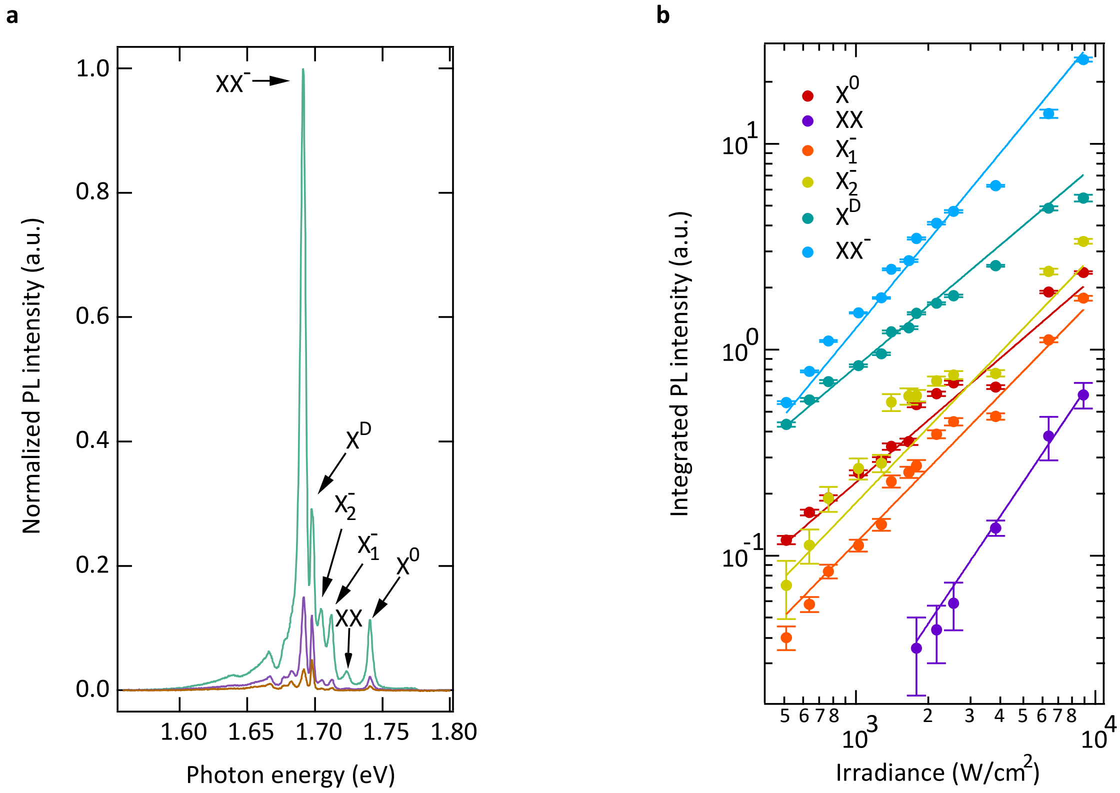

Supplementary Figure 7 | Power-dependence of the PL intensity in another WSe_2 sample. a, PL spectra of a WSe_2 monolayer for different excitation intensities (P_d = 5.0x10^2 Wcm^{-2} to 6.3x10^3 Wcm^{-2}). **b,** Logarithmic plot of the integrated PL intensity as a function of the excitation intensity. Circles represent the experimental data, while lines represent a power law fit, $\propto P^{\alpha}$, with exponents of X^0 (α = 1.01), XX (α = 1.73), X_1^- (α =1.19), X_2^- (α =1.21), X^0 (α = 0.99), XX^- (α = 1.41). The power-dependence measurements are performed without gate voltage (intrinsic regime).

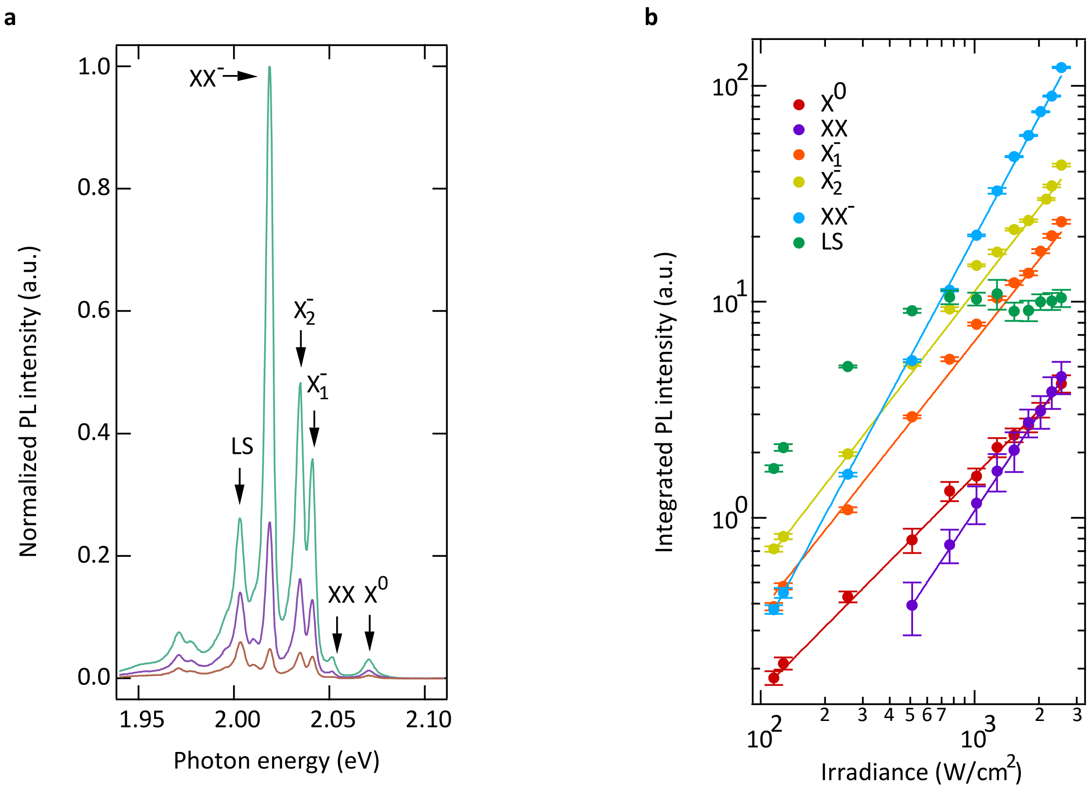

Supplementary Figure 8 | Power-dependence of the PL intensity in WS$_2$. a, PL spectra of a WS$_2$ monolayer for different excitation intensities (P_d = 5.1x10^2 Wcm^{-2} to 2.5x10^3 Wcm^{-2}). **b,** Logarithmic plot of the integrated PL intensity as a function of the excitation intensity. Circles represent the experimental data, while lines represent a power law fit, $\propto P^{\alpha}$, with exponents of X^0 (α = 1.01), XX (α = 1.49), X$_1^-$ (α = 1.25), X$_2^-$ (α = 1.28), XX$^-$ (α = 1.84). The peak LS (α = 0.2) at an energy of 2.006 eV, is most probably due to a localized state or phonon-assisted emission[11,12]. The power-dependence measurements are performed without gate voltage (intrinsic regime) on the position marked with a cross in Figure 1b of the main text.

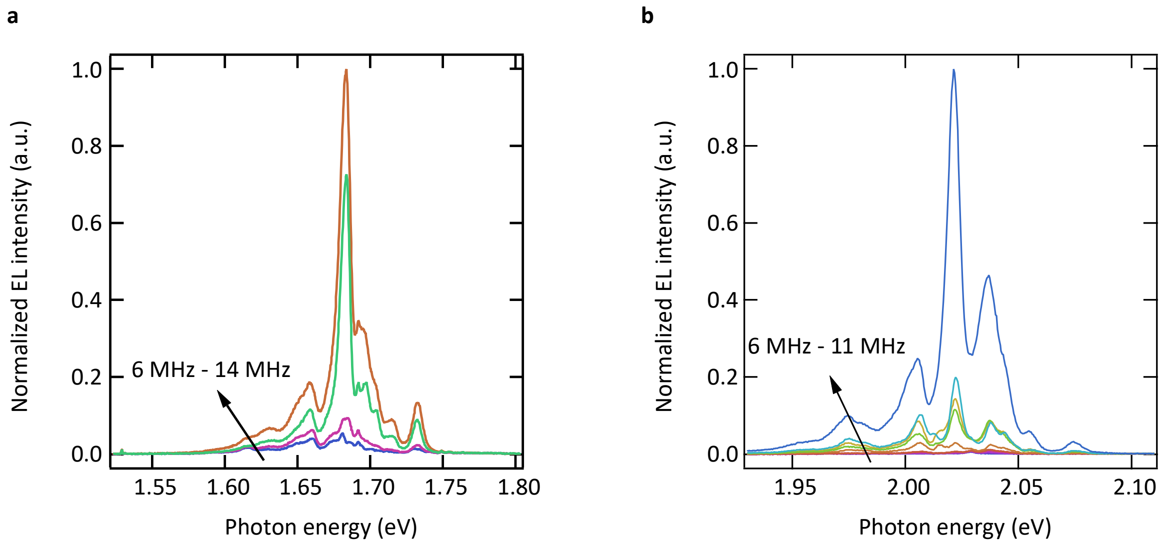

Supplementary Figure 9 | EL intensity as a function of the pulse frequency. a, WSe_2 EL spectra at frequencies ranging from 6 MHz to 14 MHz. **b,** WS_2 EL spectra at frequencies ranging from 6 MHz to 11 MHz. The emission intensity increases with frequency, in agreement with similar measurements performed at room temperature[13]. Furthermore, all the emission peaks broaden at higher frequencies.

	X^0	XX	X_1^-	X_2^-	X^D	XX^-	L_1
**WSe_2**							
EL	1	1.44	-	-	1.03	1.75	-
PL	1.01	1.82	1.19*	1.1	1.08	1.44	0.6
**WS_2**							
EL	1	2.33	1.32	1.33	-	2.31	-
PL	1.00	1.49	1.25	1.28	-	1.84	0.2

Supplementary Table 1 | α coefficient values obtained from the power-law fits in EL and PL for the different exciton species. *This value is obtained from measurements performed in a WSe_2 sample different from that presented in the main text and shown in Supplementary Figure 7.

Supplementary references

1. Ye, Z. *et al.* Efficient generation of neutral and charged biexcitons in encapsulated WSe2 monolayers. *Nat. Commun.* **9**, 3718 (2018).

2. Li, Z. *et al.* Revealing the biexciton and trion-exciton complexes in BN encapsulated WSe2. *Nat. Commun.* **9**, 3719 (2018).

3. Barbone, M. *et al.* Charge-tuneable biexciton complexes in monolayer WSe2. *Nat. Commun.* **9**, 3721 (2018).

4. Chen, S.-Y., Goldstein, T., Taniguchi, T., Watanabe, K. & Yan, J. Coulomb-bound four- and five-particle intervalley states in an atomically-thin semiconductor. *Nat. Commun.* **9**, 3717 (2018).

5. Wang, G. *et al.* In-Plane Propagation of Light in Transition Metal Dichalcogenide Monolayers: Optical Selection Rules. *Phys. Rev. Lett.* **119**, 047401 (2017).

6. Tonndorf, P. *et al.* Single-photon emission from localized excitons in an atomically thin semiconductor. *Optica* **2**, 347 (2015).

7. Srivastava, A. *et al.* Optically active quantum dots in monolayer WSe2. *Nat. Nanotechnol.* **10**, 491–496 (2015).

8. Koperski, M. *et al.* Single photon emitters in exfoliated WSe2 structures. *Nat. Nanotechnol.* **10**, 503–506 (2015).

9. Chakraborty, C., Kinnischtzke, L., Goodfellow, K. M., Beams, R. & Vamivakas, A. N. Voltage-controlled quantum light from an atomically thin semiconductor. *Nat. Nanotechnol.* **10**, 507–511 (2015).

10. He, Y.-M. *et al.* Single quantum emitters in monolayer semiconductors. *Nat. Nanotechnol.* **10**, 497–502 (2015).

11. Nagler, P. *et al.* Zeeman Splitting and Inverted Polarization of Biexciton Emission in Monolayer WS2. *Phys. Rev. Lett.* **121**, 057402 (2018).

12. Jones, A. M. *et al.* Excitonic luminescence upconversion in a two-dimensional semiconductor. *Nat. Phys.* **12**, 323–327 (2016).

13. Lien, D.-H. *et al.* Large-area and bright pulsed electroluminescence in monolayer semiconductors. *Nat. Commun.* **9**, 1229 (2018).
